# Anatomy Education in Nigeria: A Study of Program Curricula Toward Advancing Training and Improving Program Outcomes

**DOI:** 10.7759/cureus.24772

**Published:** 2022-05-06

**Authors:** Joshua Ola Owolabi, Sunday Yinka Olatunji, Adedeji E Adetunji, Tijani Adekilekun

**Affiliations:** 1 Anatomy and Neuroscience, University of Global Health Equity, Kigali, RWA; 2 Anatomy and Neuroscience, Babcock University, Ilishan-Remo, NGA; 3 Anatomy, College of Medicine and Health Sciences, Afe Babalola University, Ado-Ekiti, NGA; 4 Anatomy, Kampala International Univesity, Kampala, UGA

**Keywords:** training, career, nigeria, curriculum, education, anatomical sciences, anatomy

## Abstract

Background

Anatomy education in this context refers to the training of anatomists particularly in the university or college setting with an emphasis on equipping them with skills to be biomedical researchers and scientists, educators, and providers of applied or allied health services. There has been a recurring call to carefully evaluate and scrutinize biomedical science programs in Nigerian universities. This study considered the anatomy curriculum in representative Nigerian institutions with an emphasis on their philosophy, program design, program objectives, and program contents among other considerations.

Materials and methods

Structured and validated questionnaires, electronic, were administered to collect quantitative and qualitative data from heads of the anatomy department in representative institutions. Head of anatomy departments in 11 representative institutions returned their properly completed questionnaires, representing over 60% return rate of the target representative institutions. Quantitative data sets were analyzed and presented as tables, charts, and figures. Qualitative data in the form of free responses were analyzed and presented based on themes.

Results

Degree programs, including bachelor's, master's, and doctorate degrees, are currently offered in respondents’ universities. The curricula are generally robust in scope and depth of content as they address all the main domains of anatomy or anatomical sciences, especially gross anatomy, histology, embryology, neuroscience, and physical anthropology in many instances. The average duration for the bachelor’s program (BSc) is 4 years, master's 2 years, and PhD (Doctor of Philosophy) 3-5 years. Analysis of the main methods of training indicated that the programs include significant coursework at every level as well as the main research project leading to the presentation of a dissertation or thesis. We also identified gaps in training, with emphasis on transferable skills, which must be addressed in line with modern realities in basic medical sciences.

Conclusion

We consider it a necessity to equip graduates at all levels of training with competencies that are directly and clearly aligned with the roles that graduates of the program should play in workplaces. We, therefore, recommend that curricula be reviewed to emphasize competencies in scientific investigations, transferable skills, and science education. Specific cutting-edge skills and research methods should be included in alignment with overall program objectives and deliverables.

## Introduction

Background

This research was carried out to contribute to knowledge in the field of anatomy or anatomical science education, especially in Nigeria and Africa. This is evident from the fact that the Anatomical Society of Nigeria devoted three consecutive conferences and the annual general meetings, between 2017 and 2019, to themes and topics that revolved around anatomy education in Nigeria. It is also important to note that the body of anatomists in Nigeria was one of the foremost and largest professional basic medical science (BMS) bodies on the continent of Africa. Over the years, Nigerian institutions have trained a significant number of anatomists who work and practice within the country and many others who are providing educational and research services in institutions across the world with significant impacts. Despite the relatively significant successes that have been recorded over the years, the call for a thorough review of curricular philosophies, contents, and methods of training is in line with the global realities in medical sciences. This also needs to be done in line with the changes that have characterized education globally and more specifically BMS training. This is to ensure that the practices and the processes of training basic medical scientists are best aligned with global best practices and professional roles as determined by job requirements and governing policies of employment. To achieve this, it was clear that empirical data would be of immense benefit, hence the need to conduct this study.

About anatomy education

Need for Change and Advancements

Anatomy, the science, as well as anatomy education has no doubt evolved globally, due to the advancements in sciences generally, but more specifically due to advancements in methods of scientific investigations that have provided the avenue for a better and advanced appreciation of the human body from several perspectives [1,2]. In the early stages, anatomy was primarily concerned with the appreciation of the body's gross morphology. Over the years, there has been an increased need to explore the human body or other forms of life from gross, clinical, developmental, histological, microscopic, cellular, and subcellular perspectives. There has also been further advancement of anatomy or anatomical sciences into advanced, cutting-edge, and applied aspects, which now enables the application of anatomical science knowledge and skills in various walks of life [2]. For example, with significant emphasis on basic and applied aspects of morphological anthropology, fields such as ergonomics and forensic sciences could benefit from anatomical knowledge, skills, and applications. Furthermore, with creativity and an innovative approach to system thinking, for example, the applications of comparative and applied anatomical sciences to various aspects of design and device development could make anatomical sciences quite more relevant, arguably more than ever before. Other areas of advancements on which anatomists can leverage also include cell biology and molecular sciences using cutting-edge approaches and molecular study tools. Generally, the case for advancements in anatomy and biomedical sciences has always been made [1,3,4].

Previous Work on Training

A few studies, including ours, in the past, have attempted to examine the training regimen for anatomists, particularly in the Nigerian educational system [5,6]. Some of these studies have advocated for the need to reconsider program designs, especially in terms of skills and competencies that trainees do require by the time they complete their training. Emphasis also included a need to align such skills and competencies with demands in relevant sectors, including academia and various relevant industries that are involved in research and development activities. The need to consider applied services that trained anatomists can provide has also been emphasized [4]. For example, graduates of anatomy programs with specializations or advanced knowledge and skills in morphological sciences can significantly contribute to advancement in forensic sciences in the security industry and relevant government sectors [3]. Also, those with applied knowledge and advanced skills or specialization that include cellular and molecular biology may work in different industries where such skills are required or found to be relevant.

Previous Work on Employability

There has also been previous work that evaluated the job and career prospects, including the employability of graduates of anatomy programs at various levels including bachelor's, master's, and PhD [4,6-8]. The consensus has remained that a major factor that might limit the prospects of the program graduates, for now, might be the level of competence that these graduates might have particularly in view of the fact that industries now require skills that could enable employees to provide cutting-edge services. In line with these realities, it has, therefore, become quite important to consider the existing curricula, such that the training regimen can equip program graduates with requisite skills to make them relevant and skilled, hence employable in specific target industries or sectors [4,6-8].

*Previous Work on Policies and Practices* 

Policies and work-related practices have also been considered in relation to how graduates of anatomy programs have fared in the relevant sectors as well as factors that have influenced employability and job satisfaction among other considerations. One major factor that has stood out is the need to consider the existing policies that define the role of anatomists in the relevant sectors and workplaces. This, by extension, would also include policies that guide the practice of anatomical sciences in academia, the health sector, and other relevant industries. In the Nigerian case, for example, efforts have been made to reconsider the Anatomy Act of 1934 in line with modern realities and as such define the roles of Nigerian anatomists within their sphere of professional influence which includes educational, medical, and relevant biomedical industries as well as public offices. There is an urgent and important need to put in place proper policies and work-related regulations that will enable Nigerian anatomists to optimally contribute their professional quota toward national development [4,6-8].

A Review of Global Best Practices

The world is not only becoming more of a global village but a closer one at that which buttresses the fact that it is becoming increasingly important to consider global or international best practices with respect to program design and training regimens. In this instance, the training of anatomists is not an exception. Nigerian anatomists are no doubt robustly trained and have been found to be very adaptable and highly resourceful both in the national and international arenas [8]. There is, therefore, a need to keep up with global trends and advancements.

## Materials and methods

Mixed methods

This study used a structured questionnaire to collect both quantitative and qualitative (free response) information from the respondents. A structured and validated questionnaire was administered to the heads of departments who were required to supply information based on the design and implementation of the anatomy programs - Bachelor's, Master’s, and PhD - as applicable to their department. The initial sections, which constituted the larger part of the questionnaire, required largely quantitative data. The latter part had open-ended questions to which the heads of the departments were required to respond. These provided a suitable avenue for the respondents to provide information in qualitative formats.

Questionnaire structure

A structured and validated questionnaire on anatomical education curriculum analysis was designed based on a standard reference [9] to investigate the factors that affected the various forms, quality, and effectiveness of anatomy education in Nigeria. The carefully structured questionnaire helped to review curricula and compare the philosophies, training methods, and career prospects between representative institutions in Nigeria in order to develop practical and solution-oriented teaching and career development policies, systems, methods, and strategies for anatomists to meet the demands of Nigeria, especially in terms of research and specialized skills applications. It was logical to posit that finding these factors and analyzing their impacts and implications adequately might help to provide effective solutions that could improve students’ performances and graduates’ career prospects, thereby contributing adequately to national developments and self-actualization as professionals. The questionnaire had 10 main parts (Table 1). 

**Table 1:** Research questionnaire and its main parts.

S/N	Parts
1	Program Structure - Degree-Awarding Programs
2	Program Philosophy and Mission Statement
3	Background Information
4	Course List and Units: Undergraduate
5	Course List and Units: Postgraduate - Master's
6	Course List and Units: Doctorate
7	Program Prospects and Employability of Graduates
8	Specific Contributions to National Development
9	Program and Training-Associated Challenges
10	Free Responses: Personal Insight Into Anatomical Education in Terms of Philosophies, Training, and Prospects

Questionnaire administration

Questionnaire administration lasted a month during which respondents were required to return the properly completed questionnaire. The questionnaire was adapted to a Google form template and delivered to the target respondents through their valid email addresses. An accompanying message in the form of an email or a phone call was added to specifically request the participation of the respondents on behalf of their institutions. 

Ethical considerations

This study requested and obtained official ethical approval from Babcock University Research Ethical Committee (BUHREC: 701/18). The development, validation, and administration of the questionnaire followed all ethical considerations, requirements, and guidelines. All respondents indicated their informed consent. Data and information from respondents were all treated with strict adherence to ethical standards with respect to research data management. Responses were coded and saved anonymously. Analysis was also done without any specific references to respondents’ identities and confidential records. 

## Results

Program description

Diploma/Pre-school/Prelim

It is important to note that certain schools in the country offered pre-degree programs that might also involve certain other science disciplines, hence we decided to investigate the structure and the duration of such programs. Where applicable, the minimum duration ranged between one and two years with the average being 1^1^/_2_ years. The maximum also ranged between one and three years with the average being two years. 

*Undergraduate/Bachelor’s Programs* 

We considered the minimum and maximum stipulated durations for the undergraduate/bachelor’s programs with an emphasis on the duration. The minimum duration ranged between two and five years with the modal value being three years and the average being 3.5 years. In terms of the maximum duration, the modal value was four years, the range being 4-6 years and the average being five years; nine of the universities that participated indicated that they offered bachelor's degree programs in human anatomy. 

Postgraduate - Master's

The minimum duration for the master’s program ranged between one and three years with the modal value being two years. The maximum duration ranged between two and three years with the average being 2.5 years. Out of 13 universities, four indicated that they did not offer master’s degree programs. 

Postgraduate - Doctorate

The minimum duration for the doctorate program (PhD or Doctor of Philosophy) was generally three years. The maximum duration ranged between four and five years with the average being five years. Out of 13 universities, four indicated that they did not offer doctorate programs. The nomenclature of the doctorate was also always a Doctor of Philosophy - PhD, in anatomy.

Postgraduate - Postdoctorate

Only five respondents indicated that they had a place for postdoctoral training. The duration indicated for a postdoctoral training is one year generally. 

Program philosophy and mission statement

**Figure 1:**
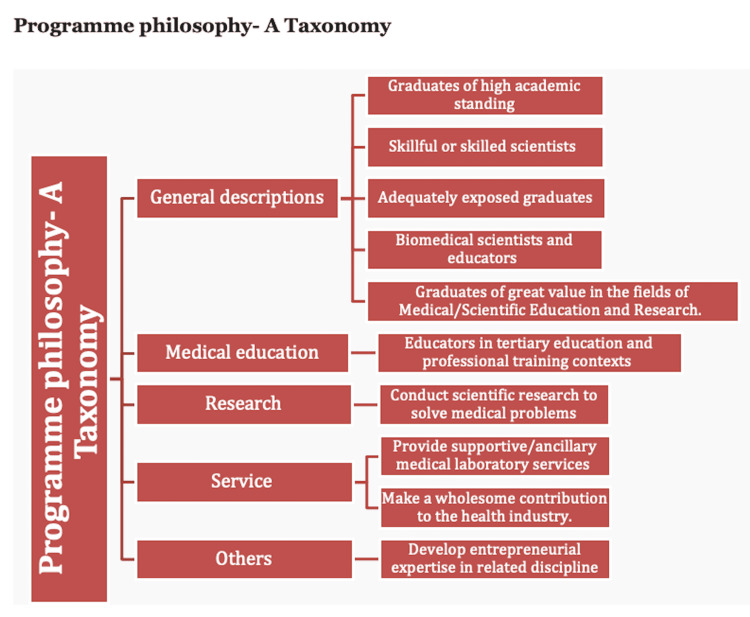
Program philosophy as indicated by participants organized as a taxonomy. The statement or description of program philosophy could be grouped under themes including general descriptions, medical education, research, services, and others. Following the determination of the key themes, representative statements were placed appropriately under the themes.

**Figure 2:**
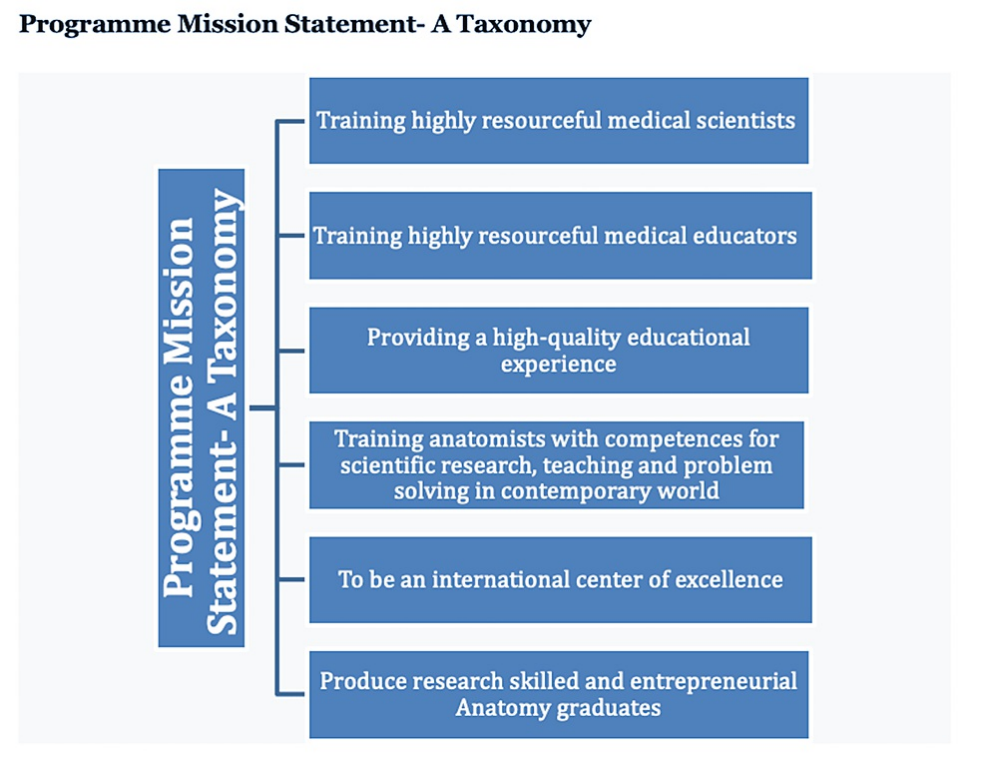
A taxonomic illustration of the themes that academic leaders gave, based on their anatomy curricula about their mission statement. The most emphasized this being about training competent and resourceful medical scientific and educators. These two were specific to the awareness while others were generic, placing emphasis on departments and institutions.

In terms of philosophy and mission statement, Nigerian institutions trained anatomists as medical scientists, with primary prospects of working as medical scientists and educators, providing services primarily in the educational sectors, including universities and other institutions that offer anatomy, medical, and allied health programs. They are also trained to provide services that might require their competencies as medical scientists in the relevant industries. In both program philosophy (Figure 1) and mission statements (Figure 2), the most predominant themes and terms included "medical scientists" and "educators" - these, therefore, would primarily define Nigerian anatomists. 

Structure of programs

Curriculum review showed that anatomy, as offered in the Nigerian context, was primarily human anatomy, as a basic medical or biomedical science program (Table 2). Significant core course content is integrated into programs at every level. The programs also included a significant proportion of non-anatomical BMS content as well as non-medical science content. The programs always included a major project leading to the presentation and defense of a dissertation or thesis. Anatomy programs at all levels are essentially full-time programs (Table 3). The major job opportunities included teaching in the universities, nursing schools, and similar institutions of health sciences well as medical scientists in non-academic settings. Working in the civil service was ranked with the least prospects (Table 4). There is a unanimous position as 100% of academic leaders that participated indicated that anatomists should be employed as medical scientists, researchers, and university teachers/lecturers. Notwithstanding, responses showed that the curricula could train medical scientists with employability opportunities and prospects in several other sectors such as paramedics, civil servants, other tertiary institution teachers, and even high school teachers (Table 5). Poor career recognition, limited job opportunities, and career prospects including limited training facilities topped the list of challenges facing anatomy programs (Table 6).

**Table 2:** Background information on the anatomy program, especially in terms of the overall description and context of the program.

		Yes%	No%	NA%
	Anatomy offered as a Bachelor's Degree Course/Programme	90.9	9.1	0.0
	Anatomy offered as Human Anatomy	90.9	9.1	0.0
	Anatomy offered as a Basic Medical Science Degree	90.9	9.1	0.0
	Anatomy offered as a Biological Science Degree	0.0	100.0	0.0

**Table 3:** Information on course units and breakdown for anatomy degree programs with emphasis on the estimated overall units, the core courses' units, and estimated units allocated to other courses. BMS, basic medical science.

	Average Total Units	Average Core Anatomy Units	Average Non-Anatomy BMS Units	Average General Knowledge/Competences Courses Units	Anatomy/Medical Education	Average Project Units
BSc	155	75	26	25	19	6
MSc	81	62	37	27	26	7
PhD	39	36	3	2	6	10

**Table 4:** Information about program prospects and employability of graduates, allowing respondents to rate the probability of their programs’ graduates to get employment in the specific relevant sectors/quarters on the scale of 0-3; where 0 = no, 1 = low, 2 = moderate, and 3 = high.

		0	1	2	3
1	Employment in the Health Sectors as Paramedics	9.1	0.0	54.5	36.4
2	Employment in the Health Sectors as Service providers and Health Assistants Employment in the Civil Services	0.0	18.2	54.5	27.3
3	Employment in Non-educational Institutes/Organizations as Researchers and Medical Scientists	0.0	9.1	18.2	72.7
4	Employment in the University as Lecturers	0.0	0.0	27.3	72.7
5	Employment in Other Tertiary Institutions (e.g. Nursing Schools) as Teachers/Instructors	0.0	9.1	9.1	81.8
6	Employment as Secondary/High School Teachers	18.2	9.1	27.3	45.5

**Table 5:** Respondents' positions about the specific contributions of their anatomy programs as every level applicable (BSc, MSc, PhD) to national development. BSc, Bachelor of Science; MSc, Master of Science; PhD, Doctor of Philosophy.

		Yes%	No%	NA%
1	Employment as Paramedics	90.9	9.1	0.0
2	Employment as Civil Servants	81.8	18.2	0.0
3	Employment as Researchers and Medical Scientists	100.0	0.0	0.0
4	Employment as University Lecturers	100.0	0.0	0.0
5	Employment as Other Tertiary Institution Teachers	81.8	18.2	0.0
6	Employment as Secondary/High School Teachers	72.7	27.3	0.0

**Table 6:** Respondents' position on factors that are typical program challenges in their settings or contexts.

		Yes%	No%	NA%
1	Limited Training Facilities	72.7	27.3	0.0
2	Limited Students’ Interest in the Course of Study	63.6	36.4	0.0
3	Limited Job Opportunities and Career Prospects	81.8	18.2	0.0
4	Poor Career Recognition	90.9	9.1	0.0
5	Outdated Curriculum Content	18.2	72.7	9.1

## Discussion

Program description

The program duration, based on the information obtained during this study, shows that the average duration for a bachelor's program in anatomy ranges between four and five years. This is in line with the regulations of the National University Commission of Nigeria, in which case a degree program will run between three and five years. The option of having a degree program within three years is only possible if the entrant would come in with advanced A-Level qualification or an ordinary national diploma (OND) from an appropriate institution such as the polytechnic. The conventional duration is therefore four years, while under certain circumstances such as in the universities of technology, the standard program duration could be five years due to their policies and institutional standards. This duration, in line with global best practices, will be adequate to obtain a bachelor's degree from a university. It is also important to note that the structure of the program requires that students are trained with adequate depth in all primary domains including knowledge, skills, and attitude. With such program durations for the anatomy programs, it is evident that there would be adequate time for not just knowledge acquisition but also quality research. What, therefore, becomes equally important is the need to emphasize research while training students or trainees during these stipulated durations.

Generally, respondents indicated that the program is offered in human anatomy, and in the context of BMSs. A critical look at the course contents might indicate extensions and expansions into comparative and applied anatomy. However, the core course contents revolve around human anatomy in the context of biomedical science or BMS, whereas the domains, phenomena, and bodies of knowledge that involve interactions between the human body milieus with other life-forms such as microorganisms, the plant kingdom as well as lower forms of life even within the animal kingdom are considered. These observations, altogether, therefore point to the fact that the programs are typically very robust, offering the graduates the versatility and potential to apply their knowledge of anatomy and skills to a very broad range of fields of endeavor. This equally points to the need to expand the career prospect for graduates, especially in terms of policies, career programs, and orientation.

The minimum duration for the master's program range between one and three years while the maximum range is between two and three years. It is also clear that this is in line with global best practices especially since the program structure incorporates both theoretical courses as well as practical training and research skill acquisition in addition to a comprehensive research project leading to the development and defense of a thesis or dissertation. The minimum duration for the PhD is generally placed at three years with a maximum range between four and five years. This is also in line with global best practices, noting that this in many instances also includes training and teaching that include theoretical concepts, practical application of theories as well as specific research skills and competencies. It is, therefore, clear that the duration is adequate to provide students with the opportunity to be grounded in all the required domains. What has become, therefore, very important for emphasis is the need to utilize this duration optimally to acquire quality competencies especially as it relates to integration into communities of practice and learning. While this was not specifically explored, it is important to emphasize the need to integrate postgraduate trainees into communities of practice and learning to continue their professional growth and development through quality exposure, professional networking, and collaborations. Poor quality of supervision has been a bane of doctorate programs. In response, there have been programs and recommendations, including the need to adopt creative and effective approaches to students’ supervision mentorship [10]. Few of the respondents indicated that the training duration for postdocs was generally put at one year. However, based on background information, the postdoctoral training program is another area that needs attention not just in Nigeria but in Africa in general. There is a need to seize the early years following the acquisition of a PhD or doctorate for mentoring fresh doctorates and to help them acquire requisite expertise in their chosen fields and indoctrinate them into the world and business of science and scientific investigations. The benefits of postdoctoral training cannot be overemphasized, and this is also in line with global best practices.

Program philosophy and mission statement

A common theme that runs through the respondents’ statements in terms of the mission statement and philosophy of the program included the fact that students who enroll in their anatomy programs are trained as medical scientists and educators. It is also clear that this is in line with the mainstream philosophy of why programs in anatomy or biomedical sciences are offered in Nigerian universities [11]. Many graduates of the program proceed to obtain higher degrees and qualifications, and subsequently become teachers in medical schools or schools of health sciences. This would further imply that they contribute to the training of several health professionals such as medical doctors, medical laboratory scientists, nurses, pharmacists, public health professionals, and other medical scientists. Expectedly, anatomists who become educators are also expected to conduct research. It is important to emphasize the fact that not all medical scientists are expected to be educators, rather some others might choose to become biomedical scientists providing relevant services in industries that are primarily outside the academic domains. It is, therefore, important to appreciate the fact that certain anatomists would leave academia and proceed almost entirely to build careers as scientists and investigators in various Industries. The most relevant industries may include pharmaceutical industries, biopharma industries, health product industries, as well as industries that provide services that might include but are not limited to forensic services, and assisted reproductive technology services, among others. This should influence the training regimen, such that anatomy educators who train anatomists in academia can offer them insights, exposure, and training opportunities that prepare them for such jobs, services, and roles. For anatomists who become anatomy and medical educators primarily, there is a need for their training to emphasize concepts in the fields of anatomy, medical, and health science education, with particular emphasis on the major pillars such as curriculum, pedagogies, assessments, academic leadership, program evaluation, and educational technologies or innovations [12-16].

Notably, there is an important industry that appears to be undervalued in terms of how much emphasis is laid on the potential contributions of anatomists. This observation also underscores the need to consider the roles of anatomists beyond the teach-train-and-do-research roles in mainstream academia of tertiary institutions. This industry includes the education industry as it bothers with the provision of educational services and the developing of educational materials. For example, anatomists who proceed to become neuroscientists, which is typical in Nigeria and Africa, can contribute significantly to the educational neuroscience field by exploring concepts such as human memories and learning methods and mechanisms, and by promoting the application of knowledge and evidence to support evidence-based policies and practices. Another vital example is the development of educational materials such as anatomical and medical illustrations. Being anatomical and medical illustrators is another example of services that an anatomist might provide. More than ever before, the need to produce and provide very accurate anatomical, medical, and scientific illustrations is being emphasized. In fact, this goes beyond the artistic drawings, to include dynamic illustrations as well as three-dimensional (3D) representations of the human body systems, organs, and structures as well as concepts and phenomena. Anatomists who serve as anatomical, medical, and scientific illustrators will, therefore, be needed in the educational industries either as practicing illustrators or as specialists who oversee illustration projects, validating representations, and providing consultancy services. It is also important to emphasize that beyond the university, anatomists can also provide health science education teaching to pre-university students such as A-level or high school or college pupils, especially with additional training or competence in health science education. 

Several industries might need applied anatomical knowledge and skills, such as in the aspects of ergonomics and system designs. This might range from the clothing and fashion industries to the automobile and computer technology industries, and anatomists could provide expert guidance and design guidelines based on human morphology, kinetics and gestures, postures and behaviors on visualization, fitness-for-use, comfort-related design considerations, and movement and body-shape-related design considerations. While the basic training for an anatomist might not offer all the requisite skills and competencies, it remains fundamental and foundational, such that the applied training and skills can help to provide such services. The training regimen should, therefore, consider these realities in terms of its philosophy, approach, and methods.

It is interesting to note the relatively consistent undertone that defines the kind of laboratory services in terms of anatomists’ roles as merely supportive or ancillary. It is, however, important to emphasize that while this might be true in certain instances, the anatomist, being trained as a medical or biomedical scientist, can always serve in a principal role in relevant laboratories. This undertone might be a residual mindset from a historical background that places certain categories of health professionals and scientists in ancillary categories. This line of thought has so far become obsolete. There remains a vital need to disabuse this mindset as it is important to emphasize that with adequate training biomedical scientists when qualified do serve in principal roles while providing relevant laboratory services. It is also important to further emphasize that every trained anatomist, as a biomedical scientist, is capable of providing quality laboratory services in academic laboratories, and when qualified and certified in specific relevant service laboratories. Stakeholders should be aware of this and should take responsibility for correcting this anomaly that is typically observable and could be prevalent in certain instances. 

Structure of programs

In terms of structure, all programs including bachelor's, master's, and doctorate include both significant theoretical learning as well as practical and applied training. It is important to note that this is in line with the prevalent philosophy of training in the country and Africa in general [11]. Graduates are expected to have not just the core knowledge and skills, but they are also required to apply their knowledge in specific domains. It is also important to note that the training, while emphasizing the core concept, also includes training in areas that are related to anatomical sciences in other walks of life where knowledge of anatomical science could be applicable. It is important to note that anatomy or anatomical science in the context of training in Nigeria incorporates the traditional and emerging fields of anatomy. The core subdisciplines include gross anatomy, histology and histochemistry and embryology, genetics, and other fields that are related to these disciplines. Several applied fields such as assisted reproductive sciences and technology, forensic sciences, and physical anthropology have also been incorporated into the training. This is important for emphasis as anatomy in certain other climes has been reduced to either gross anatomy or clinical anatomy which is basically gross anatomy in a clinical or applied context. Very significant benefits of this approach for the Nigerian anatomist include the fact that they are grounded in all branches of anatomical sciences and might choose to specialize in any of the branches. The training also provides a solid and broad base for career development. When it might be important to optimize and sustain this approach, it is equally important to train anatomists in certain advanced, specialized subdiscipline areas such that graduates are skilled and can become experts in such advanced domains [11]. 

Personal and professional insights

**Figure 3:**
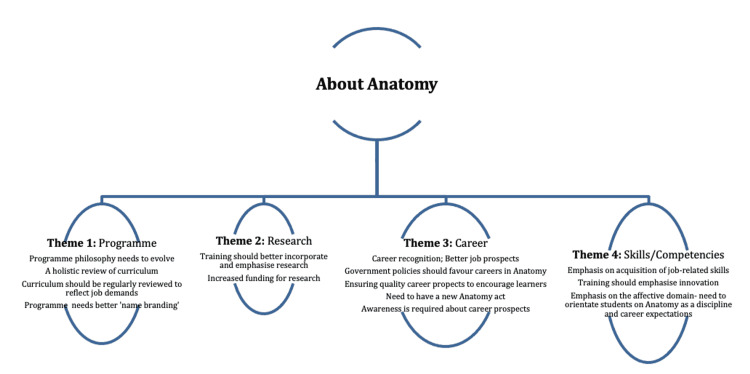
Free responses of academic leaders and heads of anatomy about their personal insight into anatomical education in terms of philosophies, training, and prospects in Nigeria and Africa, toward improving training and career prospects to meet the needs of the Nigeria and the Africa continent.

The free responses of participants were thematically analyzed. The key themes (see Figure 3) include an emphasis on the program, research, career, and skills/competencies of the program graduates. Overall, participants were largely of the belief that the current status and situation need a significant positive change. This includes the need to redesign the curriculum starting with the most fundamental consideration, the philosophy. This would imply that the "essence" and "purpose" of the program need to be redefined in line with the current realities not just at the national level but also at the global level. Clearly, there are traditional career roles of anatomists; however, there are also emerging roles to which the curriculum should respond [17]. Noting that the respondents were academic leaders and heads of departments, their suggestions about the need for a program redesign would suggest that they saw this need as being relatively beyond their capacities. This is not unconnected with the fact that there are concerned government agencies that are involved in primarily defining programs and setting benchmarks for training. One would, therefore, posit that change should start at such levels. Another level is the institutional level, where institutional leaders should heed the call of professionals and academic leaders and provide avenues for not just dialogs and resolutions but also actions that could help to revolutionize anatomy as a discipline, and, by extension, BMSs. If this is done properly, it is clear that the curriculum would meet the needs of trainees in terms of how best to prepare them for the modern job market and societal realities. Career prospects' emphasis is of local and global interest in the field of medical sciences, and this is, therefore, no exception [18].

The research was also highlighted. Respondents believed that training programs should better emphasize cutting-edge research, not just in curricular philosophy but also in training. A vital consideration would be that trainees should acquire adequate transferable research skills. Also, to enshrine a culture of research, adequate funding should be provided to equip laboratories and drive engagements in cutting-edge research. Respondents also emphasized the need to ensure better job prospects for program graduates. It is noteworthy that curricular philosophies generally indicated that program graduates would be medical scientists, educators, researchers, and service providers in relevant industries and sectors. As such, recognition should be given to anatomists in association with those defined roles. It was believed that this would motivate trainees. It was also believed that awareness for both learners and the public would be vital to promoting better career recognition and prospects. Therefore, the need for training to emphasize research skills and applied competencies remains a major consideration.

Respondents generally believed that there is a critical need to equip trainees with skills in the areas of biomedical sciences research, education, scientific services, and roles among others. This has also been previously reported [14]. It was also believed that there should be a set of clearly identified competencies that graduates of anatomy programs should have to prepare them for the employment market. This is in line with global trends as much as it is in line with advancements in biomedical sciences in general [19,20]. The need to consider innovations and technological applications in the medical sciences was also emphasized.

Limitations

The main limitation of this study was that we could not extend the qualitative aspects to include in-depth interviews and all focus group discussions due to logistics constraints including limited availability of resources, time, and funding.

## Conclusions

In conclusion, the Nigeria Anatomy Program is very robust in philosophy and indicative content. It is, therefore, important to ensure a process that optimizes the training programs based on the curricula. It is necessary to equip graduates at all levels of training with competencies that are directly and clearly aligned with the roles that graduates of the program should play in workplaces. It is, therefore, recommended that the program delivery should emphasize competency in scientific investigation and science education. Specific cutting-edge skills and research methods should be included in alignment with overall program objectives and deliverables. 
